# Immune Responses Accelerate Ageing: Proof-of-Principle in an Insect Model

**DOI:** 10.1371/journal.pone.0019972

**Published:** 2011-05-18

**Authors:** E. Rhiannon Pursall, Jens Rolff

**Affiliations:** 1 School of Biosciences, University of Birmingham, Birmingham, United Kingdom; 2 Department of Animal and Plant Sciences, University of Sheffield, Sheffield, United Kingdom; University of Pittsburgh, United States of America

## Abstract

The pathology of many of the world's most important infectious diseases is caused by the immune response. Additionally age-related disease is often attributed to inflammatory responses. Consequently a reduction in infections and hence inflammation early in life has been hypothesized to explain the rise in lifespan in industrialized societies. Here we demonstrate experimentally for the first time that eliciting an immune response early in life accelerates ageing. We use the beetle *Tenebrio molitor* as an inflammation model. We provide a proof of principle for the effects of early infection on morbidity late in life and demonstrate a long-lasting cost of immunopathology. Along with presenting a proof-of-principle study, we discuss a mechanism for the apparently counter-adaptive persistence of immunopathology in natural populations. If immunopathology from early immune response only becomes costly later in life, natural selection on reducing self-harm would be relaxed, which could explain the presence of immune self-harm in nature.

## Introduction

Inflammation has long-lasting health implications. Tissues express the inflammatory response following trauma or infection, providing the innate immune system's first response to insult. In the short term, inflammation constitutes a vital response to immune injury as it is the key component of tissue repair and reinstates physiological homeostasis [1]. However the initial acute inflammatory response can develop into a chronic condition if tissue health is not restored or irritation to the immune system is maintained [2]. Exposure to inflammation has therefore been linked to many diseases (reviewed in [3]), in which cases the inflammatory process can cause more harm than the initial immune insult [4]. In fact, the severity of many of the world's most important infectious diseases can largely be attributed to the immunopathology of inflammation [5]. In addition, diseases caused by inflammation are often associated with ageing, when disease symptoms are not expressed until many years after the initial inflammatory response [6]. These include cardiovascular disease [7], cancer [8] and metabolic diseases [3].

Recently Caleb Finch and Eileen Crimmins [9], [10] proposed that a reduction in lifetime exposure to infectious disease and subsequent reduced inflammation causes a decline in age-related adult disease, i.e. a direct link between immunopathology encountered in early life and a decrease in adulthood morbidity and mortality. Most studies supporting this hypothesis have been of epidemiological, correlational nature, and lack hypothesis-driven experimental evidence. Experimental animal studies provide very powerful means by which to investigate the mechanisms controlling how environments in early life impact on phenotype expression in human adulthood [11], [12]. As insect immunity also causes inflammation and immunopathology [13], [14], we have conducted a proof-of-principle study using the mealworm beetle *Tenebrio molitor*. The similarities between the underlying mechanisms of the insect and human innate immune systems [15] have encouraged and confirmed the importance of, the use of insect model systems in immunology. In *T. molitor* it has been shown that an immune response leads to the damage of vital organs, Malpighian tubules, which function equivalent to the kidneys of vertebrates [16].

Moreover, during the pupal phase of development in *T. molitor* a substantial proportion of the body is reconstructed: almost all larval tissues get completely dissolved and remodelled. Most of the gut is reconstructed during metamorphosis and shows different larval versus adult morphologies, but the Malpighian tubules are an exception and remain as intact structures [17], [18], [19], [20]. This allows us to exclude damage to other tissues as an underlying cause of ageing phenotypes if the timing of the treatment shortly before or after pupation does not affect aging rate. Taken together, we have a model system where inflammatory damage to an organ system has been demonstrated [16] and because the study was designed to specifically test the impact of inflammation experienced either before or after complete metamorphosis, we can investigate damage to Malpighian tubules as a candidate mechanism.

Here, we investigate whether experimental activation of immune response early in life affects ageing measured as maximum lifespan. To measure the costs of immune activation and inflammation without any concurrent cost of infection, we activated the immune system at an early life stage with two non-infectious antigens: heat-killed bacteria (*Serratia marcescens*) and nylon filament, both of which elicit an immune response [21], [22]. Animals were challenged either at the larval stage, 70 days post-hatching, or the adult stage, 8 days post-adult-eclosion. The procedural controls received an injection with sterile insect ringer, and full controls were not subjected to any immune injury. We analysed maximum lifespan to specifically measure aging [23], [24], and also medium lifespan.

## Results

### Maximum lifespan

Early-life immune response accelerated ageing, as shown by a reduction in maximum lifespan. The maximum lifespan value for each experimental group was below that of the Control group (Table 1). Each group had a different maximum lifespan as the 90^th^ percentile of survival time was calculated for each group separately.

**Table 1:** 90^th^ percentile survival times for each experimental group.

Treatment Group	Maximum Lifespan: 90^th^ Percentile Survival Time (Days)
Control	224
Procedural Control 1	219
Procedural Control 2	218
Nylon Larval Stage	205
Nylon Adult Stage	198
Bacteria Larval Stage	195
Bacteria Adult Stage	193

(Groups are listed in descending order of maximum lifespan).

**Figure 1:**
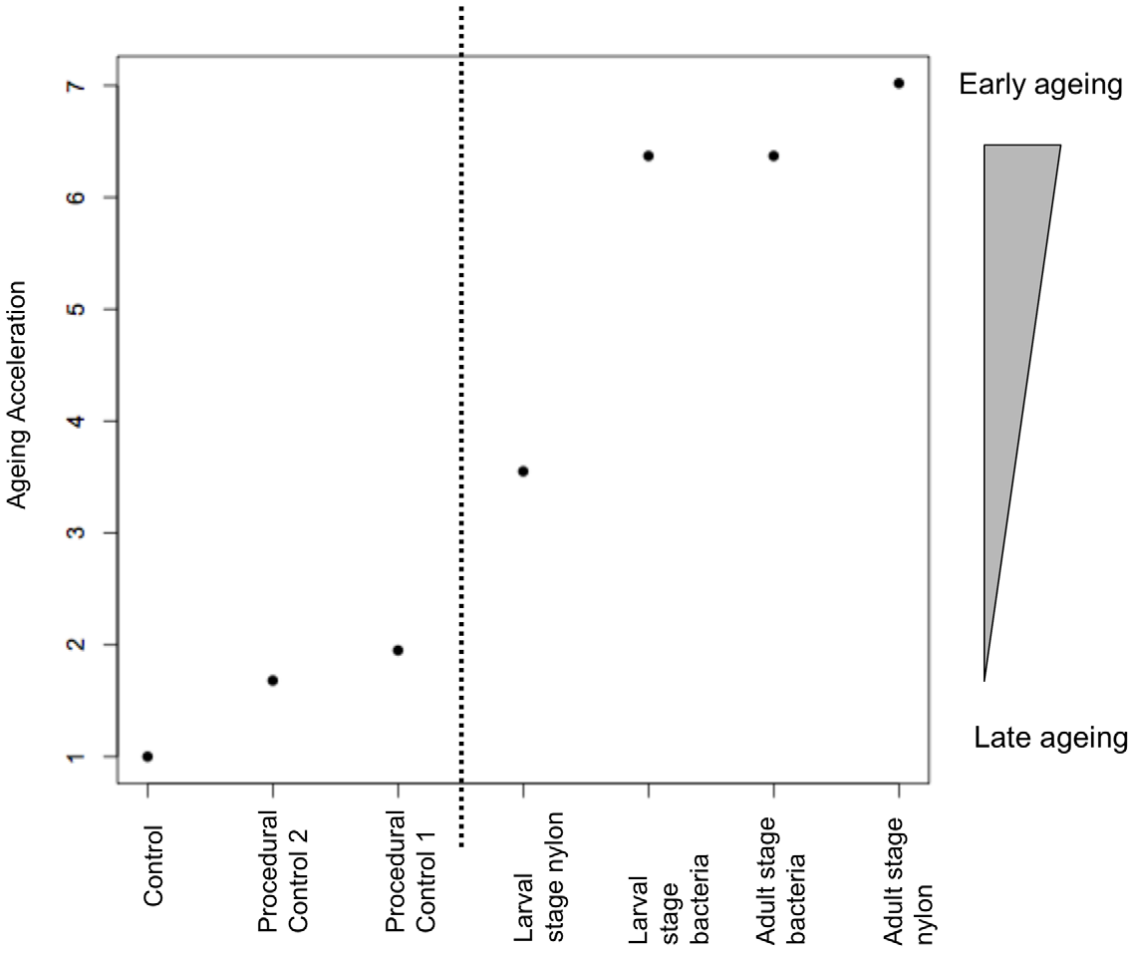
Early-life immune challenge accelerates ageing. ‘Ageing acceleration’ on the y-axis represents the reduction in maximum lifespan caused by the treatments (the percentage of survivors to the 90^th^ percentile in the control group/the percentage of survivors to the 90^th^ percentile in the treatment group). The dashed line represents the point at which the difference in aging acceleration between Control and experimental groups becomes statistically significant. Control n = 133; Procedural Control 1 n = 69; Procedural Control 2 n = 62; Nylon Larval Stage n = 97; Bacteria Adult Stage n = 29; Bacteria Larval Stage n = 58; Nylon Adult Stage n = 32. Experimental groups were as follows: Control = no experimental manipulation in larval or adult stage; Procedural Control 2 = injection with sterile insect ringer in larval stage only; Procedural Control 1 = injection with sterile insect ringer in both larval and adult stage; Larval stage nylon = insertion of nylon filament in larval stage, and injection of sterile insect ringer in adult stage; Larval stage bacteria = injection of dead bacteria in larval stage, and injection of sterile insect ringer in adult stage; Adult stage bacteria = injection of sterile insect ringer at larval stage and injection of dead bacteria in adult stage; Adult stage nylon = injection of sterile insect ringer in larval stage and insertion of nylon filament in adult stage.

In order to test for differences in maximum lifespan between experimental groups, the 90^th^ percentile of overall survival time when all groups were combined were calculated. This value was 219 days. The percentage of each group reaching this maximum lifespan was compared to the percentage of the control (Fig. 1). An Exact Unconditional z-pooled test, which is argued to be more powerful for comparing two populations than the standard unconditional tests (Berger, 1996), was used to compare percentages. All experimental groups that had been immune challenged early in life exhibited significantly shorter maximum lifespan than the control group (percentage of individuals reaching 219 days was significantly lower, P<0.03 in all cases, Table 2). Pooling all immune challenged groups, i.e. larval or adult challenge with either nylon or bacteria, and comparing them to the pooled procedural control, showed a significant effect of immune challenge on shortening maximum lifespan (Exact Unconditional Independence Test: Test Statistic = −2.60, P = 0.01). The effect of accelerated ageing was seen in all immune-challenged groups, independent of whether the immune response was elicited before or after pupation. Differences between groups challenged either before or after pupation were non-significant (P>0.5 in all comparisons).

**Table 2:** Maximum lifespan analysis using Exact Unconditional Independence (z-pooled) test.

Comparison	Treatment Effect	Test Statistic	Exact Unconditional (z-pooled) P-value
Control vs. PC1	1.68	−1.54	0.128
Control vs. PC2	1.95	−1.78	0.077
Control vs. NLS	3.55	−3.28	0.002
Control vs. BLS	6.37	−3.18	0.003
Control vs. BAS	6.37	−2.32	0.028
Control vs. NAS	7.02	−2.47	0.017

Treatment effects and p-values for comparing each group against Control, in terms of the proportion of individuals reaching 90^th^ percentile of overall survival time (219 days). Treatment groups: PC1 = Procedural Control 1; PC2 = Procedural Control 2; NLS = Nylon Larval Stage; BLS = Bacteria Larval Stage; BAS = Bacteria Adult Stage; NAS = Bacteria Adult Stage.

### Median lifespan

**Figure 2:**
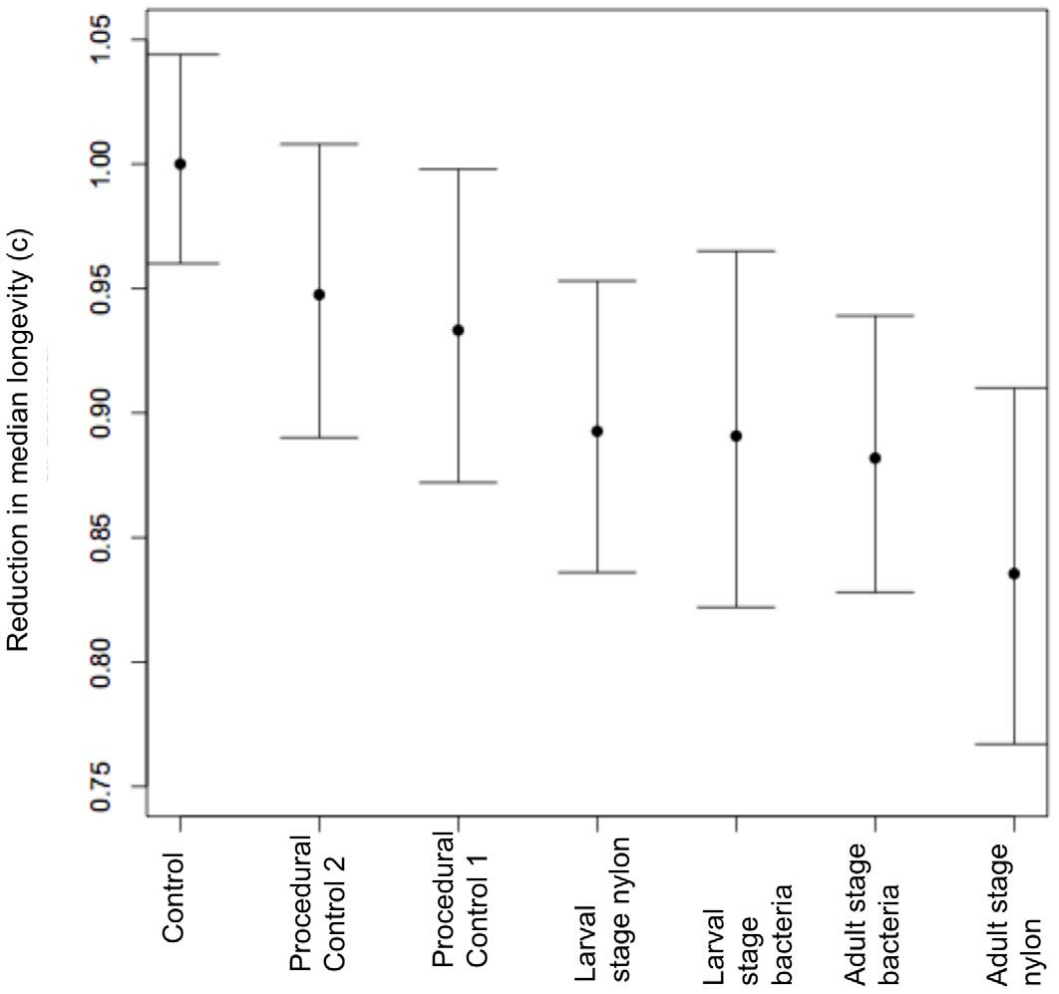
Early-life immune challenge reduces median longevity. Each c-parameter represents the percent difference in lifespan when each experimental group is compared with the control group, for any survival quantile. Error bars represent 95% confidence intervals.

The analysis of median lifespan using the accelerated failure time model showed the same overall directional pattern as maximum lifespan – full controls had the longest lifespan, followed by both procedural control groups, and the four immune challenged groups suffered the shortest lifespan (Fig. 2, table 3). The c-parameter, which is a treatment effect averaged among all survival time quantiles (i.e. the shift of the survival curve), was consistently lower when experimental treatments were compared against the control (Fig. 2). As a reference point, a treatment effect was calculated when control is compared against itself, and this value was the highest on the y-axis, confirming that early-life immune response initiates a reduction in median lifespan. Confidence intervals for these treatment effects did not overlap with the intervals of the control ‘reference point’ treatment effect (Table 3).

**Table 3:** c-Parameter estimates and confidence intervals from AFT model. Data are displayed in descending order of c-Parameter.

Group	c-Parameter	Lower CI	Upper CI
Control	1.000	0.96	1.044
Procedural Control 1	0.948	0.890	1.008
Procedural Control 2	0.933	0.872	0.998
Nylon Larval Stage	0.893	0.836	0.953
Bacteria Adult Stage	0.891	0.822	0.965
Bacteria Larval Stage	0.882	0.828	0.939
Nylon Adult Stage	0.836	0.767	0.910

In the overall pooled comparison, procedural controls did not differ from immune challenged groups in median lifespan (Exact Unconditional Test: test statistic = −0.02, P = 0.49), unlike in maximum lifespan. However, compared to full controls there was significantly reduced median lifespan in individuals treated with either the control injection or immune antigens when all immune challenged and procedural control groups were pooled together (Chi-Squared Test: χ^2^ = 13.68, P = 0.0002).

### Physiological condition in mid-adult life

**Figure 3:**
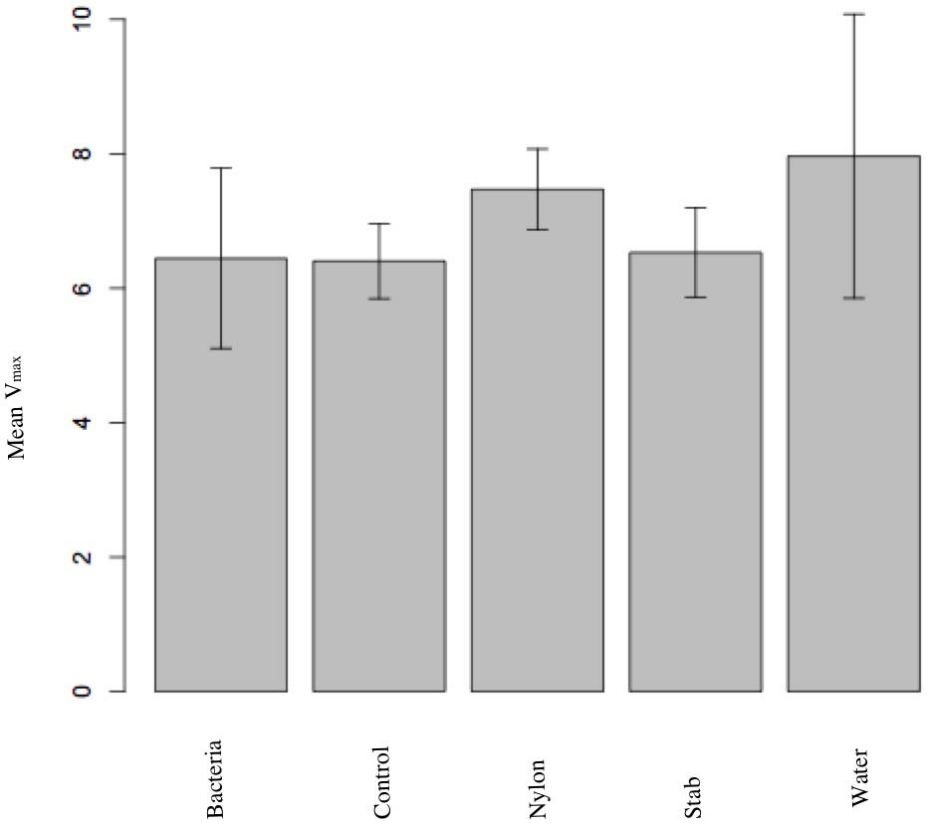
Mean haemolymph phenoloxidase activity of different experimental groups, measured as mean Vmax. Error parts convey standard errors. Bacteria, n = 23; Control, n = 76; Nylon, n = 51; Stab, n = 33; Water, n = 23.

In a study that closely mirrored the experimental procedures of the main study presented here, we asked if the results could be driven by a resource trade-off. Our data do not show an indication of such a trade-off as levels of the enzyme phenoloxidase in the mid-adult stage were not affected by early-life immune challenge (ANOVA: F = 0.5727; df = 4; P = 0.6828; Fig. 3).

## Discussion

The same ageing phenotype – a reduction in median and maximum lifespan - is observed when an immune insult is generated by a variety of different antigens, either before or after metamorphosis. This proof-of-principle that early-life inflammation causes reduced adult survival is important for two types of study that have covered this topic; firstly, clinical experiments indicating links between inflammation and adult disease [3], [7], [25], [26], [27], [28], [29] and secondly, studies showing that birth cohorts experiencing high levels of early-life mortality also experience reduced lifespan [6], [30], [31].

We also investigated the notion that the aging phenotypes could be caused by a resource trade-off investigating the role of phenoloxidase, a crucial component of the innate immune system. Briefly, this is an enzyme involved in two major processes; i) the catalisation of the oxidation of tyrosine to 3,4-dihydroxyphenylananine; and ii) melanisation [32]. It also has been demonstrated to be condition dependent ([33]). Our results indicate that immune challenges did not cause phenoloxidase to be upregulated in the long term. This is in concordance with previous experimentation showing that *Tenebrio molitor* immune response ceases 28 days following infection [34].

The phenoloxidase response is associated with tissue damage in *Tenebrio molitor* [16]. Since phenoloxidase levels in our study did not appear to be upregulated in the long-term, we can confidently hypothesise that the observed lifespan reduction was not caused by repeated tissue damage from persistent phenoloxidase. Instead, we suggest that immunopathological damage suffered at the time of inflammation and expressed later in life is the parsimonious explanation for our findings. In support of this the experimental procedure elicited an immune response without a living pathogen. Moreover, the effects of immune response were delayed, with the first mortality detected 64 days following infection (Figure S1).

Further evidence to support our hypothesis that immunopathology is an important mechanism behind the long-term cost of early-life inflammation comes from our investigation into the timing of infection. Eliciting immune response before or after complete metamorphosis did not affect the results. This may suggest that inflammation-associated tissue damage contributing to lifespan reduction is carried through complete metamorphosis. In support of this, Malpighian tubules have been shown to be particularly sensitive to immunopathology in *T. molitor*, suffering reduction in functioning [16]. Malpighian tubules function as insect ‘kidneys’, and are among the organs of the insect body that are not completely reconstructed during metamorphosis from larva to adult [19], [20].

We propose that an initial immune response and upregulation of phenoloxidase could have inflicted harm on Malpighian tubules that persisted into adult life and caused reduced lifespan. If this were to indicate a general evolutionary mechanism, it is possible that the link between childhood inflammation and reduced lifespan observed by Finch and Crimmins [9] could be driven by immunopathological damage incurred during inflammation, the long-lasting effects of which are carried through to adulthood.

Whilst our data are consistent with the notion that early inflammation leads to increased aging mediated by innate responses, the question remains if this observation relates also to vertebrates. Vertebrates have a more complex immune system, the acquired immune system, yet inflammation and immunopathology in vertebrates has also been shown to be partly mediated by NF-kB mediated innate responses. The latter are highly conserved and form an important part of insect immunity ([35], [36], [37].

In an evolutionary context, we would like to argue that immunopathology could play a role in organisms in the wild. Costs of immune responses become exacerbated under environmental stress [38] and hence any effect on the aging phenotype will be shifted forward in an individual's lifespan and become subject to natural selection. Moreover a recent study demonstrated for the first time that insect populations in the wild senesce, exhibiting age-dependent survivorship [39]. Alternatively, immunopathology could provide an explanation for the ‘paradox of aging science’ [40]: aging (i.e. reduction in maximum lifespan) is present in most species yet it directly opposes natural selection for maintenance systems. An immune response constitutes a trade-off between the damage caused by a pathogen and self-harm. If the costs of self-harm only present at a later stage in life, and cause acceleration in aging but not a reduction in median lifespan, then natural selection on reducing self-harm will be weak.

## Materials and Methods

### Animal rearing

Pupae were collected from stock cultures maintained at 25±2°C with *ad libitum* supply of food and water. Following emergence and development to sexual maturity, adults were set up in mating pairs. The eggs from these mating events were to become experimental animals. During development, larvae were maintained at similar densities and with *ad libitum* access to food and water.

### Treatment groups

At day 70 post-hatch, larvae were taken from stock culture and placed in individual plastic cups. They were randomly assigned to one of four treatments; dead bacteria, nylon, procedural control or full control. Within these treatment groups, individuals were randomly assigned to one of two treatment stages; larval stage or adult stage. Larval stage was day 70 post-hatch; adult stage was day 8 post-adult eclosion, at which stage individuals had reached sexual maturity. Full control individuals remained in their cups throughout the treatment period. After treatment, larvae were placed back into their cups and maintained at 25±2°C with *ad libitum* supply of food and water. Age at death was recorded.

The treatment groups were as follows:

Dead bacteria in larval stage; sterile ringer in adult stage
Nylon in larval stage; sterile ringer in adult stage
Sterile ringer in larval stage, sterile ringer in adult stage
Sterile ringer in larval stage; dead bacteria in adult stage
Sterile ringer in larval stage; nylon in adult stage
Sterile ringer in larval stage; no treatment in adult stage
No treatment in either stage (full control)

### Bacterial culturing

Serratia marcescens was taken from glycerol stocks and grown up overnight in Luria broth (tryptone 10 g, yeast extract 5 g, NaCl 10 g, H2O 1l). This culture was then spread over Luria agar plates and grown overnight. A single colony from this plate was grown overnight in another Luria broth culture. This culture was then killed by heat shock in a water bath and autoclaved.

### Dead bacteria experimental treatment

Before treatment, larvae and adults were chilled on ice for 5 minutes. 5 µl of dead *Serratia marcescens* culture was pipetted onto parafilm, then taken up by a glass capillary. An incision was made in between the 3^rd^ and 4^th^ ventral abdominal sternites of experimental animals using a sterile 0.5 mm diameter needle. The bacterial culture was then expelled into the haemocoel. The dosage was approximately 3000 cells/5 µl.

### Nylon experimental treatment

Animals were chilled prior to infection, and an incision on the ventral side of the body was made, as described for the dead bacteria treatment. A 2 mm×0.16 mm piece of sterile nylon (Tenax) was inserted into the haemocoel through the incision.

### Sterile ringer treatment (procedural control)

Insect ringer (NaCl 128 mM, CaCl_2_ 18 mM, KCL 1.3 mM, NaHCO_3_ 2.3 mM) was pushed through a Sartorius Ministart RC 25 mm×0.2 µm filter. 5 µl ringer was injected using the method described for the dead bacteria treatment.

### Phenoloxidase activity 4 weeks after challenge

Tenebrio molitor larvae at the final instar stage were obtained from Livefood (www.livefoods.co.uk) and maintained in stock cultures (>100 larvae per culture) with ad libitum access to food and water. Individuals from each stock culture were randomly assigned to each experimental group (Table S1). Following experimental treatment at the larval stage, animals were placed into individual plastic cups with ad libitum access to water in the form of potato chunks. Beetles remained in these plastic cups throughout the experiment. On the day of adult eclosion, food in the form of rat chow was added to the cup and maintained at ad libitum levels. Adult-stage stab treatments were administered 8 days following adult eclosion. These stabs were intended to mimic the fact that most groups in the experiments of the main study underwent an experimental procedure in the adult stage, and therefore the results from this experiment could potentially infer information about the patterns in phenoloxidase levels that may have occurred in the main study.

### Haemolymph removal

On day 30 post-adult eclosion, beetles were chilled and then stabbed in the soft cervical membrane between the head and thorax. Haemolymph was removed directly from the stab wound using a pipette (Gilson Pipetman P20) and immediately frozen at −90°C. A minimum of 2 µl of haemolymph was collected from each beetle: if the beetle failed to yield this amount, it was discarded from the experiment.

### Measurement of haemolymph phenoloxidase activity

The phenoloxidase assay from [34] was used. Reagents and reactions were kept chilled on ice at all times prior to spectrophotometric measurement. Haemolymph was defrosted on ice and diluted to a ratio of 1∶20 neat haemolymph∶phosphate-buffered saline (NaCl 150 mM; Na_2_HPO_4_ 10 mM; pH 6.5). This mixture was then centrifuged at 12,200 rcf using an Eppendorf 5417R centrifuge. 8 µl of the supernatent was added to a well on a 96-well spectophotometer plate along with 56 µl sterile distilled water and 8 µl phosphate-buffered saline. Each well therefore contained a separate sample. L-DOPA (4 mg ml^−1^) was dissolved in sterile distilled water by mixing vigorously for at least 10 minutes. This concentration of L-DOPA has been previously shown to be a suitable concentration to use for the measurement of phenoloxidase activity in Tenebrio molitor haemolymph [34] The solution was then pushed through a Sartorius Ministart RC 25 mm×0.2 µm filter to remove any remaining particles of L-DOPA. 8 µl L-DOPA was added to each well on the plate, and the plate was immediately placed in a spectophotometer (Molecular Devices VERSAmax) and the measurement started. The specific reaction in the spectophotoeter was as follows: 60-minute kinetic reaction, with readings every 15 seconds; plate mixed by shaking before each measurement; incubated at 30°C. The rate at which phenoloxidase in each well converted to dopachrome was measured, and this enzyme activity was given in the form of Vmax, which directly correlated with the amount of phenoloxidase in the original haemolymph sample ([41]). For each sample, the reduced data was viewed in plot form on the spectophotometer, and the Vmax over the period of time where the reaction proceeded in a straight line was taken. This was done to remove distortion of the results caused by periods of lag or levelling-off.

### Statistical analyses of maximum and median lifespan

The statistical package R was used for analysis. We examined the difference between each treatment group and control in terms of median and maximum lifespan. To examine median lifespan, we ran accelerated failure time models, with each model representing a treatment group compared to full control. This analysis was based on a paper by William Swindell [23]. The distribution to use in the AFT models was decided by producing parametric survival models for each comparison, with the following distributions: Weibull, Exponential, Lognormal and Gaussian. We then used analysis of variance to test which distribution minimised Akaike's Information Criterion, and was therefore the appropriate distribution to use [42]. For each comparison of experimental and control group, the Weibull distribution was appropriate (see table S2; plots available on request). Quantile-quantile plots of the data from each AFT model indicated that the effects treatment were not always consistent over time, because the data did not form straight lines. Therefore, we can say that the c-values generated by the AFT model represent effects of treatment on survival, averaged over survival time. Each model produced a c-parameter, which represented the difference in median lifespan between the two groups, and 95% confidence intervals for this value. A treatment effect was then calculated. A value of 1 represents a c-parameter when the full control group is compared against itself. Data from accelerated failure time models are presented in table 3. To examine maximum lifespan, we calculated the 90^th^ percentile survival time when all treatment groups were combined. We then worked out the percentage of individuals in each treatment group surviving to this time. Exact unconditional tests, using a contingency table approach with the online calculator at www.stat.ncsu.edu/exact, were used to compare percentage survival for control group with each treatment group in turn. This produced a score-test z-pooled p-value for each of the seven comparisons. To obtain the treatment effect, the percentage survival of the full control group was divided by that of the treatment group in each comparison. This maximum lifespan method was based on that used by references 23 and 24 and the data are displayed in table S3.

## Supporting Information

Figure S1 **Survival curves for each treatment group.** Dotted lines represent treatment groups that differed significantly from full controls (p<0.05), when compared using a parametric survival model with Weibull distributions. Treatment groups: black solid = Control; brown solid = Procedural Control 1; grey solid = Procedural Control 2; red dashed = Bacteria Larval Stage; blue dashed = Bacteria Adult Stage; green dashed = Nylon Larval Stage; yellow dashed = Nylon Adult Stage.
(PPT)

Table S1 **Experimental groups.** See Materials and Methods Chapter for detail on experimental procedures and cohort design. ‘Larval stage’ refers to final instar before pupation; ‘adult stage’ refers to 8 days following adult eclosion. Stab and Water experimental groups are procedural controls for the two experimental groups, which are Bacteria and Nylon.
(DOC)

Table S2 **Distributions of survival models.** Analysis of variance results when each experimental treatment was compared with control, using different distributions. -2*LL value is the Akaike's Information Criterion.
(DOC)

Table S3 **Delayed effects of immune response: earliest point of overall survival time at which groups begin to differ in survival.** P-values from Chi-squared analysis using 7X2 contingency table are shown. By the 9^th^ quantile, 65 days, some experimental groups (nylon-challenged) have begun to differ significantly from other groups. Groups other than nylon, i.e. bacteria-challenged and procedural controls, did not differ significantly at day 69 (10^th^ quantile of overall survival time) (P>0.23), but these groups did suffer significantly reduced survival by day 120 (20^th^ quantile) (P<0.05).
(DOC)
