# Supplementary figures and images for: Immune Responses Accelerate Ageing: Proof-of-Principle in an Insect Model
Source: PLoS One. 2011 May 18;6(5):e19972. doi: 10.1371/journal.pone.0019972 (PMC3097213; doi:10.1371/journal.pone.0019972)

## Slide 1
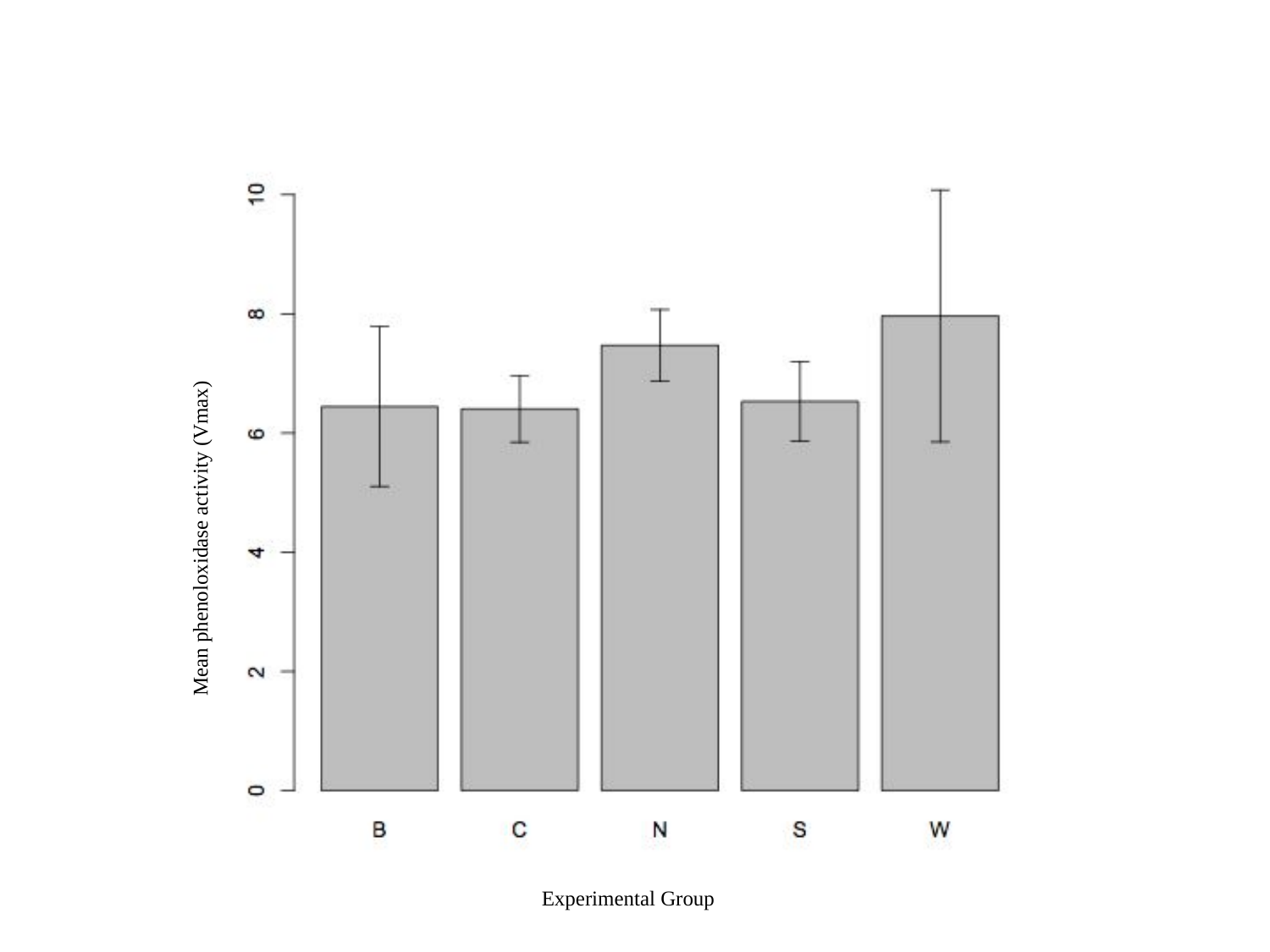

Mean phenoloxidase activity (Vmax)
Experimental Group

Supplement: Figure S1 — Survival curves for each treatment group. Dotted lines represent treatment groups that differed significantly from full controls (p<0.05), when compared using a parametric survival model with Weibull distributions. Treatment groups: black solid = Control; brown solid = Procedural Control 1; grey solid = Procedural Control 2; red dashed = Bacteria Larval Stage; blue dashed = Bacteria Adult Stage; green dashed = Nylon Larval Stage; yellow dashed = Nylon Adult Stage. (PPT) [file pone.0019972.s001.ppt]
