# Supplementary material for: Immune Responses Accelerate Ageing: Proof-of-Principle in an Insect Model
Source: PLoS One. 2011 May 18;6(5):e19972. doi: 10.1371/journal.pone.0019972 (PMC3097213; doi:10.1371/journal.pone.0019972)
Supplement: Table S1 — Experimental groups. See Materials and Methods Chapter for detail on experimental procedures and cohort design. ‘Larval stage’ refers to final instar before pupation; ‘adult stage’ refers to 8 days following adult eclosion. Stab and Water experimental groups are procedural controls for the two experimental groups, which are Bacteria and Nylon. (DOC) [file pone.0019972.s002.doc]

**Table S1. Experimental groups**. See Materials and Methods Chapter for detail on experimental procedures and cohort design. ‘Larval stage’ refers to final instar before pupation; ‘adult stage’ refers to 8 days following adult eclosion. Stab and Water experimental groups are procedural controls for the two experimental groups, which are Bacteria and Nylon.

| Group | Larval stage procedure | Adult stage procedure |
| --- | --- | --- |
| Control | No experimental treatment | Stab treatment |
| Stab | Stab treatment | Stab treatment |
| Water | Water injection | Stab treatment |
| Bacteria | Dead bacteria injection | Stab treatment |
| Nylon | Nylon injection | Stab treatment |
