# Supplementary material for: Immune Responses Accelerate Ageing: Proof-of-Principle in an Insect Model
Source: PLoS One. 2011 May 18;6(5):e19972. doi: 10.1371/journal.pone.0019972 (PMC3097213; doi:10.1371/journal.pone.0019972)
Supplement: Table S2 — Distributions of survival models. Analysis of variance results when each experimental treatment was compared with control, using different distributions. -2*LL value is the Akaike's Information Criterion. (DOC) [file pone.0019972.s003.doc]

**Table S2. Distributions of survival models**. Analysis of variance results when each experimental treatment was compared with control, using different distributions. -2*LL value is the Akaike’s Information Criterion.

| Comparison | -2*LL Value from ANOVA | |  |  |
| --- | --- | --- | --- | --- |
|  | Weibull | Exponential | Lognormal | Gaussian |
| FC+DBAS | 1647.2 | 2105.5 | 1718.3 | 1669.3 |
| FC+DBLS | 1944.6 | 2479.4 | 2024.6 | 1970.8 |
| FC+NAS | 1698.8 | 2138.9 | 1761.5 | 1711.1 |
| FC+NLS | 2413.0 | 2978.0 | 2494.0 | 2429.3 |
| FC+PC1 | 2102.7 | 2623.1 | 2183.0 | 2122.1 |
| FC+PC2 | 1998.3 | 2537.0 | 2090.2 | 2026.4 |
| FC+FC | 2669.1 | 3464.4 | 2790.7 | 2711.5 |
