# Supplementary material for: Immune Responses Accelerate Ageing: Proof-of-Principle in an Insect Model
Source: PLoS One. 2011 May 18;6(5):e19972. doi: 10.1371/journal.pone.0019972 (PMC3097213; doi:10.1371/journal.pone.0019972)
Supplement: Table S3 — Delayed effects of immune response: earliest point of overall survival time at which groups begin to differ in survival. P-values from Chi-squared analysis using 7X2 contingency table are shown. By the 9th quantile, 65 days, some experimental groups (nylon-challenged) have begun to differ significantly from other groups. Groups other than nylon, i.e. bacteria-challenged and procedural controls, did not differ significantly at day 69 (10th quantile of overall survival time) (P>0.23), but these groups did suffer significantly reduced survival by day 120 (20th quantile) (P<0.05). (DOC) [file pone.0019972.s004.doc]

##### Additional file 3 – Delayed effects of immune response

**Table S3. Delayed effects of immune response: earliest point of overall survival time at which groups begin to differ in survival.** P-values from Chi-squared analysis using 7X2 contingency table are shown. By the 9^th^ quantile, 65 days, some experimental groups (nylon-challenged) have begun to differ significantly from other groups. Groups other than nylon, i.e. bacteria-challenged and procedural controls, did not differ significantly at day 69 (10^th^ quantile of overall survival time) (P>0.23), but these groups did suffer significantly reduced survival by day 120 (20^th^ quantile) (P<0.05).

| 57 Days (8^th^ Quantile) | 64 Days (8.5^th^ Quantile) | 65 Days (9^th^ Quantile) | 69 Days (10^th^ Quantile) | 120 Days (20^th^ Quantile) | 148 Days (30^th^ Quantile) |
| --- | --- | --- | --- | --- | --- |
| 0.06 | 0.04 | 0.03 | 0.02 | 0.00 | 0.00 |
